# Genome-wide open chromatin regions and their effects on the regulation of silk protein genes in *Bombyx mori*

**DOI:** 10.1038/s41598-017-13186-6

**Published:** 2017-10-10

**Authors:** Quan Zhang, Tingcai Cheng, Shengkai Jin, Youbing Guo, Yuqian Wu, Duolian Liu, Xiaomin Xu, Yueting Sun, Zhiqing Li, Huawei He, Qingyou Xia

**Affiliations:** 1 State Key Laboratory of Silkworm Genome Biology, Southwest University, Chongqing, 400715 P. R. China; 2 Key Laboratory of Sericultural Biology and Genetic Breeding, Ministry of Agriculture, Southwest University, Chongqing, 400715 China; 3 Chongqing Engineering and Technology Research Center for Novel Silk Materials, Southwest University, 2, Tiansheng Road, Beibei, Chongqing, 400715 China

## Abstract

Nucleosome-depleted open chromatin regions (OCRs) often harbor transcription factor (TF) binding sites that are associated with active DNA regulatory elements. To investigate the regulation of silk-protein genes, DNA molecules isolated from the silk glands of third-day fifth-instar silkworm larvae and embryo-derived (*BmE*) cells were subjected to formal dehyde-assisted isolation of regulatory elements (FAIRE) and high-throughput sequencing. In total, 68,000 OCRs were identified, and a number of TF-binding motifs were predicted. In particular, OCRs located near silk-protein genes contained potential binding sites for functional TFs. Moreover, many TFs were found to bind to clusters of OCRs upstream of silk-protein genes, and to regulate the expression of these genes. The expression of silk protein genes may be related not only to regulating TFs (such as fkh, Bmdimm, and Bmsage), but also to developmental and hormone-induced TFs (such as zen, eve, Br, and eip74ef). Elucidation of genome-wide OCRs and their regulatory motifs in silk protein genes will provide valuable data and clues for characterizing the mechanisms of transcriptional control of silk protein genes.

## Introduction

Open chromatin regions (OCRs) are nucleosome-depleted regions that can be bound by protein factors^1^ and can play various roles in DNA replication^2^, nuclear organization^3^, and gene transcription^4^. In eukaryotes, nucleosome disruption is a basic feature of active OCRs, which contain *cis*-elements bound by transcription factors (TFs)^5^. These *cis*-elements are conserved among eukaryotes and regulate gene expression^6^. Therefore, the characterization of OCR profiles could improve our understanding of how TFs are recognized in distinct genomic sequences to regulate target-gene transcription.

*Bombyx mori* is a model Lepidoptera species that is valuable in the sericulture industry. The larval silk gland of these insects is a specialized organ that synthesizes, assembles, and secretes silk proteins. Specifically, silk proteins such as fibroins and sericins are primarily produced in the posterior silk gland (PSG) and middle silk gland (MSG), respectively, and the genes that encode these factors exhibit rhythmic “on” and “off” transcriptional regulation during development, resulting in distinct and specific temporal and spatial expression^7^. These features make the silk gland a good model for studying transcriptional regulation networks. To date, such studies have focused mainly on how TFs regulate the expression of silk protein genes. For instance, Silk Gland Factor 2 (SGF2) is a homeodomain-containing protein that regulates the expression of the fibroin gene^8^, while, the Awh protein, which contains an LIM homeodomain, is a key regulatory factor of three fibroin genes^9^. Additionally, the juvenile hormone-TF Bmdimm was shown to be involved in the synthesis of silk proteins^10^. While each of these studies shows that particular TFs influence expression of specific genes, they do not provide comprehensive information regarding the genome-wide interactions between TFs and silk protein genes within the silkworm.

Next-generation sequencing technology can be combined with various genome-wide assays, such as self-transcribing active regulatory region (STARR)^11,12^, chromatin immunoprecipitation (ChIP)^13,14^, and nucleosome-depleted or “open chromatin” site isolation assays. In particular, these latter assays exploit DNase1 hypersensitivity sites^15^ or formaldehyde-assisted isolation of regulatory elements (FAIRE)^16^. Notably, combinations of these assays have been widely used in ENCODE and modENCODE projects for genome-wide identification of functional elements involved in gene regulation in model organisms^17,18^. In the silkworm, the first lepidopteran species to have its genome sequenced^7^, sequences upstream of silk protein genes were shown to be important for transcriptional regulation. However, the motifs in these regions were mostly predicted using genome data^7^. To further our understanding of silk protein gene regulation and associated regulatory motifs in *B. mori*, we subjected DNA isolated from the silk glands of this organism to FAIRE-seq and transcriptomic sequencing (RNA-seq) analyses; a genome-wide map of OCRs was obtained and we identified regulatory motifs for OCRs. We also sought to elucidate the relationship between regulatory motifs and the expression level of silk protein genes. Our results provide valuable data and insights, and will aid in enhancing our understanding of the mechanisms governing the specific and efficient transcription of silk protein genes.

## Results

### FAIRE-seq data

**Figure 1:**
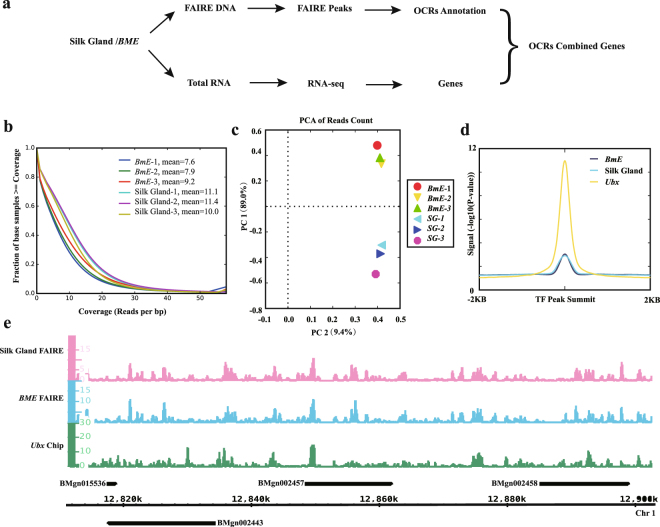
Quality control analysis of the next-generation sequencing data. (**a**) Analysis workflow. (**b**) Coverage of reads by formaldehyde-assisted isolation of regulatory elements (FAIRE). One color represents one sample. Mean is the mean coverage per bp, i.e., 20% of the sampled bp has up to 11 overlapping reads. A tiny fraction of bp had >30 overlapping reads. (**c**) Principal component analysis (PCA) plot for the FAIRE replicates of *Bombyx mori* embryo-derived (*BmE*) cells and silk glands. PC1 (89.0%) and PC2 (9.4%) are the top two principal components. (**d**) The plot of FAIRE signal at Ubx peaks. (**e**) Browser representation of the slit locus. Silk gland FAIRE, from top to bottom: silk gland FAIRE peaks, *BmE* FAIRE peaks, and Ubx chip peaks^47^.

Here, DNA molecules isolated from *B. mori* silk glands and a *BmE* cell line (Fig. 1a) were analyzed by FAIRE-seq. In total, 104,910,457 raw reads were obtained from six FAIRE-seq libraries using a Hiseq. 2000 device. After filtering out low-quality reads, 104,766,790 high-quality reads remained, with an average trimming ratio of 99% (Fig. S1), of which approximately 62,040,984 (59.2%) mapped to the silkworm genome (KAIKObase) (Table 1). The total size of the FAIRE-seq data was roughly 52 Gb. For each sample, the per-base coverage ranged from seven to 11, and approximately 20% of the reads overlapped about 11 times (Fig. 1b). We also found samples with high correlations among biological repeats and low correlations among different samples (Fig. 1c). The FAIRE signal closely resembled that of the silkworm TF BmUbx, as obtained by ChIP-seq analysis (Fig. 1d,e), thereby supporting the well-established association between nucleosome depletion and TF Ubx binding.

**Table 1:** Raw reads and mapping ratio obtained by formaldehyde-assisted isolation of regulatory elements and high throughput sequencing (FAIRE-seq).

Sample	Raw Reads	Clean Reads	Mapped Reads	Mapped Ratio
**Silk Gland 1**	17,763,376	17,751,024	12,726,420	68.76%
**Silk Gland 2**	18,442,463	18,429,266	13,387,769	69.79%
**Silk Gland 3**	20,721,593	20,707,144	15,157,617	70.65%
***BmE*** **1**	13,772,496	13,729,475	5,496,343	37.64%
***BmE*** **2**	15,639,596	15,603,680	6,928,316	41.85%
***BmE*** **3**	18,570,933	18,546,201	8,344,519	42.51%
**Total**	104,910,457	104,766,790	62,040,984	55.20%

### Peaks and OCRs

In total, 736,850 raw peaks were obtained and annotated. For each sample, the number of raw peaks ranged from 90,000 to 150,000, covering 4% to 8% of the genome (Table 2). To facilitate subsequent analyses, we merged the peaks from the biological replicates. Only peaks associated with -log10 (q-value) ≥5 and fold-change values ≥5 were considered OCRs. Accordingly, 69,519 and 41,477 confidence peaks were obtained from the *BmE* cell and silk gland samples, covering 4.1% and 3.1% of the genome, respectively (Table 2). Here, we obtained 10,246 silk gland-specific OCRs and 37,851 *BmE* cell-specific OCRs. The average length of the *BmE* cell OCRs (300 bp) was less than that of the silk gland OCRs (380 bp).

**Table 2:** Distribution of open chromatin regions (OCRs).

Sample	Raw Peaks	OCR	Specific OCRs
**Silk Gland 1**	147,967 (7.74%)	41477 (3.1%)	10,246
**Silk Gland 2**	163,362 (8.75%)		
**Silk Gland 3**	137,253 (7.05%)		
***BmE*** **1**	97,058 (4.36%)	69519 (4.1%)	37,851
***BmE*** **2**	91,336 (4.03%)		
***BmE*** **3**	99,874 (4.59%)		

OCRs were generated by merging the replicate peaks and Q-value ≥5 & fold change value ≥5.

**Figure 2:**
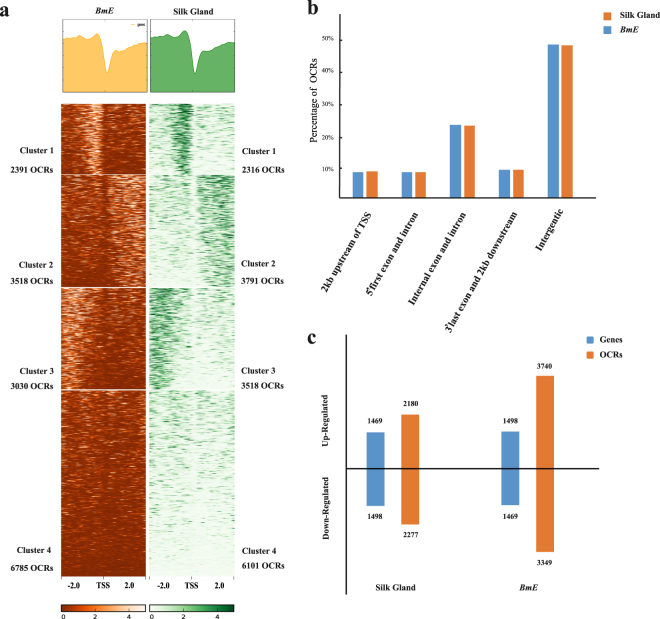
The distribution and function of OCRs. (**a**) Profile and heatmap of open chromatin regions (OCRs) of *Bombyx mori* embryo-derived (*BmE*) cells and silk glands. The distribution profile of OCRs within+/−2 kb of transcriptional start sites (TSS). Heatmap of TSS+/−2 kb region OCRs were clustered using k-means = 4. The number of OCRs in every cluster was marked in the picture both silk gland and *BmE* cell. (**b**) The genomic location of OCRs. Orange bar represents silk gland OCRs; blue bar represents *BmE* OCRs. (**c**) OCRs and regulated genes of *BmE* cells and silk glands. Up-regulated and down-regulated genes are considered those whose expression level was significantly higher or lower in the silk gland than in *BmE* cells, respectively. OCRs located within 2 kb of a gene were considered that adjacent gene’s OCR. Orange bar represents genes; blue bar represents OCRs.

Generally, OCRs are located in functional regions of chromosomes. In the silkworm, about 50% of OCRs were located in intergenic regions: nearly 17% in 3′- and 5′-untranslated regions (UTRs), approximately 8% in the promoter regions, and roughly 25% in the gene body (Fig. 2b). We subsequently plotted an OCR distribution profile spanning 2 kb upstream and downstream of the transcription start sites (TSS) (Fig. 2a); this analysis showed that the region with the highest frequency of OCRs was approximately 200 bp upstream of TSS. We also plotted an OCR signal distribution heatmap and clustered the OCRs by their distance to the TSS, with a threshold k-means = 4, and generated four groups of OCRs (Fig. 2a). Clusters 1 and 3 were comprised of OCRs located upstream of the TSS, with those in Cluster 1 being closer to the TSS than those in Cluster 3. Meanwhile, Cluster 2 contained OCRs located downstream of the TSS and Cluster 4 contained ubiquitous sites. We then combined the OCRs and their adjacent genes to reflect any information regarding OCR function to its downstream genes (Fig. 2c). Notably, OCRs which maybe have biologic function appeared in 2KB region of up-regulated or down-regulated genes in both silk gland and *BmE* cells. For instance, of the 1,469 up-regulated genes identified in the silk gland, 2,180 were located within 2 kb of an OCR. These results indicate that the transcriptional regulation of genes might be associated with the distance between the TSS and adjacent OCRs.

### OCRs harbor regulatory motifs

OCRs have been shown to harbor *cis*-elements, which are target sites of transcriptional regulatory factors^19^. In total, we identified 129 different TF binding motifs in the roughly 110,000 OCRs tested, using a threshold p-value of <1e-50. Specifically, 56 types of motif were found in 41,477 silk gland OCRs, while 54 were found in 69,616 *BmE* cell OCRs (Tables S1 and S2). In both the silk gland and *BmE* cells, 21 types of zinc finger domain TF-binding motif, 20 types of homeobox TF- binding motif, 16 types of the basic helix-loop-helix domain TF-binding motif, 9 types of basic leucine zipper domain TF-binding motif, and 3 types of FOX TF-binding motif were identified. The zinc finger domain TF-binding motif was the most frequent type observed in both the silk gland and *BmE* cells. In the silk gland, the Egr1 motif was detected in 3,901 OCRs and gave the highest significance (p-value = 1e-1087). Egr1 is a zinc-finger domain TF that is believed to function in genes associated with cell differentiation and mitogenesis^20^. In *BmE* cells, the KLF14 binding motif was the most significant site (p-value = 1e-2590) and was detected in 22,156 *BmE* OCRs. This factor is also a zinc-finger domain TF, and is a member of the Krüppel-like factor family of transcription factors that are subject to parent-specific gene expression^21^.

Additionally, we compared the motifs detected in the OCRs of the silk gland and *BmE* cells (Fig. S2, Table S3) and identified seven silk gland-specific motifs. Notably, nearly all of these motifs were HOX TF-binding motifs. Thus, these HOX motifs comprise tissue-selective motifs in fifth instar silkworm larva. Meanwhile, we also detected five *BmE* cell type-selective motifs. The types of motifs were diverse, including TATA-box and Runt motifs.

In this study, we detected 75 *de novo* motifs, 39 from *BmE* cells and 36 from the silk gland (Tables S1 and S2). After annotation, we found that many of these motifs were similar to known motifs. For example, Motif 9_SG was similar to *Drosophila melanogaster* Br-Z3, which is involved in hormone responses^22^. Additionally, Motif 21_*BmE* highly resembled ftz (p = 1e-7016), a homeobox TF that functions in determining neuronal identity^23^.

### Regulation models of TFs

**Figure 3:**
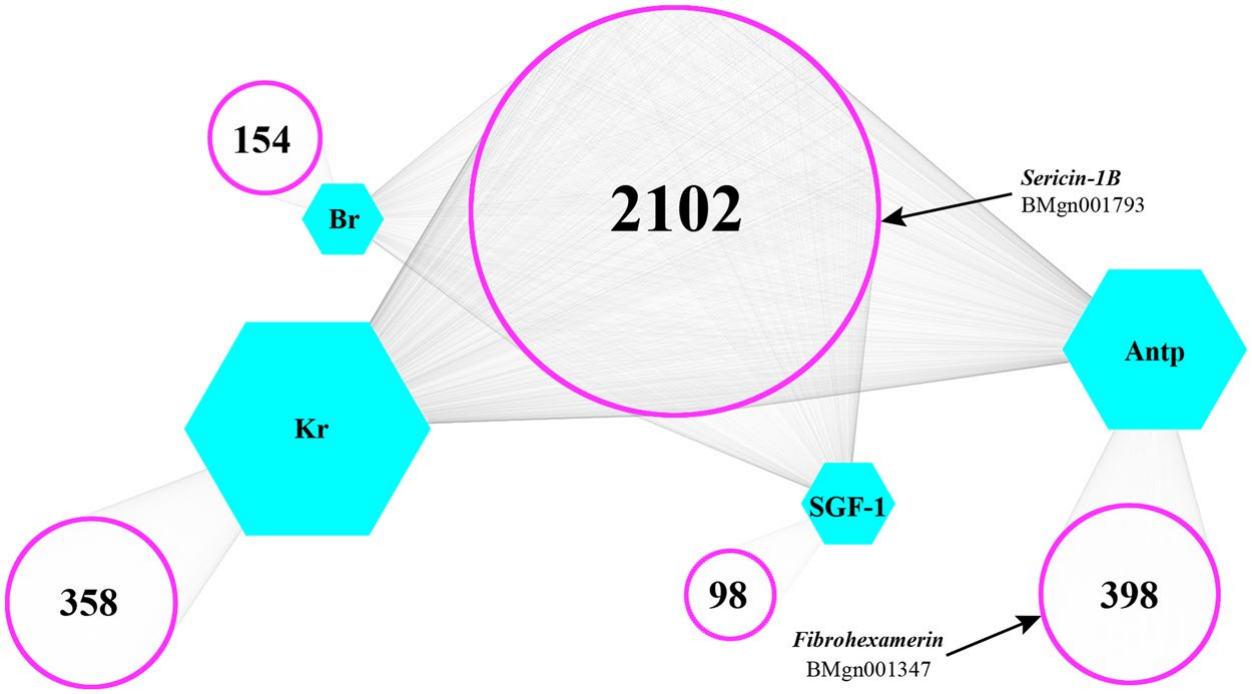
Network graphic for four transcription factors (TFs) and their adjacent genes. Hexagons represent known motifs. The points that form the purple circles represent genes regulated by the corresponding TF. Lines are used to indicate genes that might be regulated by the corresponding TFs. Genes located within the big circle located in the middle might be regulated by two or more TFs. Genes within the peripheral small circle might be regulated by only one TF.

The motifs described above may be bound by some common TFs. We listed several TFs that exhibited differential expression levels between the silk gland and *BmE* cells (Table 3) and assigned the adjacent genes of their binding motifs as targeted genes. Four TFs (Br^24^, Kr^25^, Antp^26^, and SGF-1^27^) were selected to build gene regulatory models (Fig. 3). In total, 3,111 genes were discovered within 10-kb regions surrounding the four TFs. Of these, 2,102 (67%) exhibited regulation by more than one TF. For example, an SGF-1 binding motif was located 441 bp upstream from *Sericin-1B*, and Br- and Kr-binding motifs were located 712 bp upstream from *Sericin-1B*. These three TFs could therefore co-regulate *Sericin-1B*. In addition, 1,099 (33%) genes showed regulation by a specific TF. For example, an Antp-binding motif was found 2,853 bp upstream from *Fibrohexamerin*. This regulation model could be valuable for the identification of potentially new relationships between TFs and genes.

**Table 3:** Silkworm transcription factor (TF) motifs detected in open chromatin regions (OCRs).

TF name	Sequence Logo	TF Structure	Gene ID	FPKM	
				Silk Gland	*BmE*
**Awh**		HTH	BMgn003888	8.965	0.000
**Antp**			BMgn006391	12.160	0.030
**Bmdimm**		bHLH	BMgn007303	258.705	0.170
**Bmsage**			BMgn005217	124.470	0.835
**Br-c Z4**		Zinc-Finger	BMgn009907	1.685	2.155
**Kr-h1**			BMgn003160	4.515	0.060
**Fkh/SGF-1**		FOX	BMgn005101	64.260	0.035

Gene ID indicates the TF-encoding gene. FPKM value denotes the expression level of the TF-encoding gene.

### The complex regulation of silk protein genes

**Figure 4:**
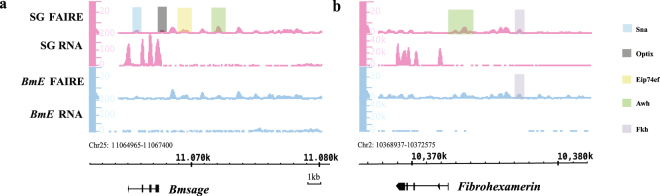
The transcriptional regulation mode of *Bmsage* and *Fibrohexamerin* gene. Formaldehyde-assisted isolation of regulatory elements (FAIRE; 0–30) and RNA (*Fibrohexamerin* 0–50,000, *Bmsage* 0–200) signals for 10-kb upstream regions from (**a**) *Bmsage* and (**b**) *Fibrohexamerin*. Pink peaks are silk gland FAIRE-seq and RNA-seq peaks. Blue peaks are *BmE* FAIRE-seq and RNA-seq peaks. Other colored shadows show locations of known transcription factor binding sites, and each color corresponds to a particular transcription factor.

Fifth-instar silkworm larvae show high levels of silk protein gene expression. Of these, *Fib-H* was expressed at a high level in the silk gland, but not in *BmE* cells (Table S3). Bmsage is a TF that was previously found to up-regulate *Fib-H* ^28^. Interestingly, the gene encoding Bmsage was also highly expressed in the silk gland, but showed almost no expression in *BmE* cells (Fig. 4a, Table 3). We therefore examined the 10-kb region upstream of *Bmsage* in both the silk gland and *BmE* cells and detected eight and four OCRs, respectively. Four of the silk gland-specific OCRs harbored the binding motifs for the TFs Awh, sna, optix, and eip74ef^29,30^, respectively. In particular, the gene encoding Awh showed higher expression in the silk gland, compared to *BmE* cells (Table 3). Sna and optix play a role in development^31,32^, whereas eip74ef was found to respond to ecdysone^33^. These data therefore suggest that regulation of silk-protein gene expression is a complex process and is associated with development and hormone responses.

*Fibrohexamerin* was also highly expressed in the silk gland (FPKM = 15716.9, log_2_ fold change = 12.6578), compared to *BmE* cells (FPKM = 2.43215) (Fig. 4b). While binding motifs for SGF-1 were detected in an OCR 6-kb upstream of the *Fibrohexamerin* gene in both the silk gland and *BmE* cells, an Awh-binding motif was detected only in the OCR of the silk gland. Similar to *Awh*, *SGF-1* expression was higher in the silk gland than in *BmE* cells (Table 3). Thus, these findings suggest that SGF-1 and Awh maybe play a role in regulating *Fibrohexamerin* transcription.

## Discussion

FAIRE-seq is an efficient method for genome-wide mapping of OCRs^34^. In the present study, we perform FAIRE-seq using samples from the silk glands of silkworm larvae and *BmE* cells, generated a map of genome-wide OCRs, discovered motifs present in these OCRs, and analyzed the regulation modes of TFs and their target genes, especially for the silk protein genes. Silk protein genes are only expressed at high levels in fifth-instar larvae^7^. Silk fibroin genes (e.g., *Fib-H* and *Fib-L*) are mainly expressed in the PSG, whereas silk sericin genes (e.g., *Sericin-1* and *Sericin-2*) are expressed in the anterior silk gland (ASG) and MSG. In this study, the six silk protein genes *Fib-H*, *Fib-L*, *Fibrohexamerin*, *Sericin-1*, *Sericin-2*, and *Sericin-3* were highly expressed in the silk gland but not in *BmE* cells. The spatial-temporal pattern of silk protein genes may be related to the distribution of adjacent OCRs.

Our results show that the signal strength and locations of OCRs are different between the silk gland and *BmE* cells. For silk protein genes, 13 OCRs were identified together within a 10-kb upstream region of their respective TSS, and five of these OCRs were silk gland-specific (Fig. S3a). Moreover, regulation of gene expression does not appear to always be mediated through a single OCR, but on occasion involves a cluster of OCRs. Clusters of open regulatory elements (COREs) have been suggested to act on the expression of genes, and particular OCRs within a CORE may have specific functions^19^. In particular, *Sericin 1* was reported to be specifically expressed in the MSG, and several studies reported the presence of various potential TF-binding motifs within approximately 4 kb of the *Sericin 1* promoter that are capable of upregulating *Sericin 1* transcription^35^. In our study, we found an OCR in the promoter region of *Sericin 1* in both the silk gland and *BmE* cells (Fig. S4a). Notably, however, we detected higher levels of *Sericin 1* expression in the silk gland than in *BmE* cells. Two silk gland-specific OCRs were subsequently identified in the silk gland *Sericin 1* gene. These OCRs in the promoter region combined with the two OCRs in the gene might therefore constitute a silk gland-specific CORE that can promote *Sericin 1* expression within the silk gland.

OCRs contain many *cis*-elements that can be bound by a variety of TFs. By analyzing the relationship between the locations of TF binding motifs and their adjacent genes, it is feasible to model gene regulation. In our dataset, an *E-box (CANNTG)* motif was found in an OCR located in the second exon of the *Fib-H* gene of the silk gland, but not of *BmE* cells (Fig. S4b). This *E-box* was deemed to comprise a binding motif for the TF Bmdimm^36^, which was expressed at a high level in the silk gland (Table 3). Given that previous studies reported that Bmdimm can up-regulate expression of *Fib-H* ^36^, our data suggest that this TF binds regulates *Fib-H* expression in third-day fifth-instar silkworm larvae via binding to the *E-box* motif.

The *Fibrohexamerin* gene was also expressed at a higher level in the silk gland, compared to *BmE* cells. Based on previous reports and the results we obtained, we predicted that *Fibrohexamerin* expression might be regulated through more than one regulatory model. In one model, *Fibrohexamerin* was regulated by Awh alone within the silk gland via binding to its motif located in the *Fibrohexamerin* promoter region. Another possibility is that Awh and SGF-1 together up-regulate *Fibrohexamerin* expression in the silk gland, but not in *BmE* cells due to the lack of an Awh motif (Fig. S4e). Meanwhile, there also appears to be a mechanism of negative regulation of OCR to gene. Specifically, we detected a ftz-binding motif in an OCR located in a region 1 kb downstream of the *Fib-L* TSS in *BmE* cells (Fig. S4c). Ftz was reported to reduce the level silk protein gene transcription^37^; thus, this factor might be involved in inhibiting the expression of *Fib-L* in *BmE* cells, but not in the silk gland. In any case, these findings indicate that transcriptional regulation of silk protein genes is a complex process.

Previous studies reported that *Fib-H* expression is co-regulated by two TFs, Bmsage and SGF-1^38^. Our data also provided some clues regarding the regulatory cascade of TFs by OCRs. For example, SGF-1-binding motifs were detected upstream of the *Bmdimm* gene in both the silk gland and *BmE* cells (Fig. S4d). It is therefore feasible that *Bmdimm* is be regulated by SGF-1. Notably, although the same three OCRs containing SGF-1-binding motifs were present in the region upstream of *Bmdimm* in both the silk gland and *BmE* cells, *Bmdimm* was expressed at high levels in the silk gland but only barely in *BmE* cells (Fig. S4d). A similar expression pattern was observed for *SGF-1* gene (Fig. S4f). We therefore inferred that SGF-1 expression might be a primary reason for the silk gland-specific expression of *Bmdimm*. Moreover, binding motifs for the TFs sna and br were also found specifically in the upstream region of *Bmdimm* in the silk gland (Fig. S4d). Thus, these factors might also play a role in this process.

Characterization of OCRs is crucial to enhancing our understanding of the relationship between TFs and target genes. Here, we sought to elucidate the mechanism of transcriptional regulation of silk protein genes. From our analysis of the chromatin regions of silk protein genes, we identified new potential regulatory sites, as well as novel modes of regulation. The relationship between TFs and target genes was explained using information from previous bioinformatics studies, and served to increase our understanding of transcriptional regulation within the silkworm. However, the information regarding the mode of silkworm transcriptional regulation obtained in this study was limited due to a lack of systematic and comprehensive data. We therefore aim to gradually address this gap in our knowledge in our follow-up studies.

## Methods

### Sample preparation

*BmE* cells were cultured in Grace medium (Gibco, Gaithersburg, MD, USA) supplemented with 10% fetal bovine serum in 75 cm^2^ cell culture canted-neck flasks (14831; Corning, Corning, NY, USA). Four flasks containing approximately 1 × 10^7^ *BmE* cells each were prepared for FAIRE-seq and RNA-seq analyses.

Three healthy silkworm larvae of the Dazao strain were chosen from the colony maintained at the State Key Laboratory of Silkworm Genome Biology. The left silk gland of each larva was used for FAIRE-seq, with each larva serving as a biological replicate; RNA-seq was carried out using the right silk glands.

### FAIRE-seq

FAIRE was carried out as described previously^16^. Briefly, silk gland tissue was ground into powder in liquid nitrogen and suspended in 1 M PBS. The powder was then treated with formaldehyde (37%) to induce crosslinks in the DNA. After quenching the activity of the formaldehyde via the addition of 125 mM glycine, the cell and tissue lysate was sonicated to achieve an average DNA fragment size of approximately 300–400 bp (7 × 30 pulses of 1 s duration, followed by 3 s of rest, at 21% amplitude) (Fig. S5). FAIRE DNA was twice extracted via the phenol–chloroform method, and the resulting aqueous layer was incubated with a one-tenth volume of 3 M sodium acetate (pH 5.2), two volumes of 95% (v/v) ethanol, and 1 μl of 20 mg·ml^−1^ glycogen. The FAIRE DNA was then digested with protease K and DNase free RNase enzyme overnight. The ratio of the concentration of FAIRE DNA (which includes only open chromatin regions) to that of the FAIRE control DNA sample (whole genomic DNA) was used as the threshold. Only samples with a threshold lower than 0.05 were used for sequencing library construction (Table S5).

Sequencing libraries were prepared according to the manufacturer’s protocols provided with the TruSeq NanoDNA Library Preparation kit (Illumina, San Diego, CA, USA). The FAIRE-seq library was initiated using 100–450 ng ofFAIRE DNA. Two rounds of purification were then performed using Agencourt AMPure XP beads (Agencourt Biosciences Co., Beverly, MA, USA); the DNA samples were then amplified using 18 PCR cycles. The amplified DNA was size-selected to 200–500 bp and sequenced using the Hiseq. 2000 system (Illumina). We found that a minimum of 1 × 10^6^ aligned reads provided robust sequencing.

### Data analysis

FAIRE-seq data was analyzed as previously described^16^, using FastQC software (Version 0.11.1). Quality control was performed with the FASTX-tool kit (Version 0.0.13) (Fig. S1), and clean reads were mapped to a reference genome (KAIKObase) by using the Burrows-Wheeler Aligner (BWA) (Version 0.7.1). Biological replicates were then merged using samtools (Version 1.4). MACS2^39^ was used for peak calling, and peak signal normalization and Pearson correlations (PCC) were performed using Deeptools2^40^ (Fig. S6). Bedtools (http://bedtools.readthedocs.io/en/latest/) was used to combine the FAIRE-seq and RNA-seq dataset. The FAIRE peaks and RNA-seq signals were all visualized with IGB (Version 9.0.0). Both the raw FAIRE-seq and RNA-seq data presented in this publication were deposited in the NCBI Short Read Archive (http://www.ncbi.nlm.nih.gov/sra/) and are accessible through SRA accession number: SRP100811.

### Motif discovery

Motif discovery was carried out using MEME (http://meme-suite.org/), RSAT^41^ (http://www.rsat.eu), and Homer (Version 4.8) software. Motifs present in all OCRs were identified by Homer (http://homer.salk.edu/homer/), while motifs in OCRs adjacent to silk protein genes were identified by MEME^42^ (Fig. S3). Short motifs without gaps were identified by DREME^43^ and similar motifs were predicted by TOMTOM^44^. Motifs were annotated using the GoMo tool (http://meme-suite.org/tools/gomo). The network graphic used for motifs was generated by RAST^41^. In the signal silk protein regulation mode analysis, we only used the report generated by JASPAR; the relative profile score was greater than 95%. Any motif with a score >9 was defined as a confidence motif.

### RNA-seq

Total RNA was isolated using the SV Total RNA Isolate System (Z3100; Promega, Madison, WI, USA), following the manufacturer’s instructions. All RNAs were screened using an Agilent 2100 Bioanalyzer (Agilent Technologies, Santa Clara, CA, USA) to ensure that the quality of the samples was sufficiently good for RNA-seq library preparation. Each RNA-seq sample yielded 0.1–1 μg total RNA, which contained enough mRNA for library establishment. The rRNA was cleaned using RNAClean XP beads (Illumina) and subjected to reverse transcription to obtain cDNAs. After adenylating the 3′ ends and ligating the adapters, the library was enriched via PCR. The average read size of the library was 260 bp, and greater than 1 × 10^6^ aligned reads were obtained.

RNA-seq data were analyzed using standard methods^45,46]^. The quality of the raw and processed reads was evaluated using FastQC (Version 0.11.1). PolyA tails were filtered by fqtrim (Version 0.93), and reads were aligned to the silkworm tRNA and rRNA database (http://www.silkdb.org/silkdb/doc/download.htm) using bowtie2 software (Version 2.2.9). Low-quality reads were removed using Trimmomatic^46^ (Table S5). The cleaned short reads were mapped to the silkworm reference genome (KAIKObase) with TopHat (Version 2.0.12.). Differentially expressed genes (DEGs) were detected using Cuffdiff^45^ and RSEM (Version 1.2.29). We defined the intersects of the DEGs generated by these two software programs as the final DEGs (Fig. S7, Table S6). Up-regulated genes were considered those whose expression level was distinctly higher in the silk gland than *BmE* cells. Conversely, down-regulated genes were considered those whose expression was markedly lower in the silk gland than *BmE* cells. For identification of up-regulated genes and down-regulated genes, they were calculated by Cuffdiff and RSEM software separately. Then, the intersection point for the Cuffdiff and RSEM was the final up/down-regulated genes (Fig. 2c).

Gene ontology (GO) enrichment analysis provides all GO terms that are significantly enriched among input genes, compared to the background genome (http://www.silkdb.org/silkdb/doc/download.html), and identifies corresponding biological functions. Indeed, this analysis is frequently used to identify the main biological functions for DEGs. Here, GO enrichment analysis was performed using OmicShare tools, a free online platform for data analysis (www.omicshare.com/tools) (Tables S7 and S8, Figs S8, S9, S10). First, all input genes were mapped to GO terms in the Gene Ontology database (http://www.geneontology.org/); gene numbers were calculated for every term, and significantly enriched GO terms for input genes, compared to the background genome, were defined via a hypergeometric test. The calculated p-value was subjected to false discovery rate (FDR) correction, with a FDR threshold of ≤0.05. GO terms meeting these conditions were defined as significantly enriched.

## Electronic supplementary material

Supplementary Information

Supplementary Tables
