# Supplementary material for: Genome-wide open chromatin regions and their effects on the regulation of silk protein genes in Bombyx mori
Source: Sci Rep. 2017 Oct 10;7:12919. doi: 10.1038/s41598-017-13186-6 (PMC5635003; doi:10.1038/s41598-017-13186-6)
Supplement: Supplementary file 1 — Supplementary Information [file 41598_2017_13186_MOESM1_ESM.pdf]

Supplemental Information

Genome-wide open chromatin regions and their effects on the regulation of silk protein genes in *Bombyx mori*

Quan Zhang[1,2], Tingcai Cheng[1,2,3]*, Shengkai Jin[1], Youbing Guo[1], Yuqian Wu[1], Duolian Liu[1], Xiaomin Xu[1], Yueting Sun[1], Zhiqing Li[1], Huawei He[1,2,3], Qingyou Xia[1,2,3]

1. State Key Laboratory of Silkworm Genome Biology, Southwest University, Chongqing 400715, P. R. China

2. Key Laboratory of Sericultural Biology and Genetic Breeding, Ministry of Agriculture, Southwest University, Chongqing, 400715, China

3. Chongqing Engineering and Technology Research Center for Novel Silk Materials, Southwest University, 2, Tiansheng Road, Beibei, Chongqing, 400715, China.

*Correspondence author. Address: State Key Laboratory of Silkworm Genome Biology, Southwest University, Chongqing 400715, China

Tel: +86 23 68250099; Fax: +86 23 68251128

E-mail address: chengtc@swu.edu.cn (Cheng T)

Contents:

Supplementary Tables:

Supplementary Table S1. Silk Gland OCRs harbored Motifs (Known and De nove) summary

Supplementary Table S2. BmE OCRs harbored Motifs (Known and De nove) summary

Supplementary Table S3. Silk Gland and BmE specific motifs

Supplementary Table S4. RNA-seq quality control and differentially expressed genes (DEGs)

Supplementary Table S5. FAIRE DNA quality

Supplementary Table S6. Different expressed genes between BmE and Silk Gland

Supplementary Table S7. Silk Gland up-regulated gene GO annotation

Supplementary Table S8. BmE up-regulated gene GO annotation

Supplementary Figures:

Supplementary Figure S1. Quality scores of all base

Supplementary Figure S2. Wenn graph of silk gland and BmE specific motif

Supplementary Figure S3. Location and annotation of new discovered motifs adjacent to 6 silk protein genes.

Supplementary Figure S4. FAIRE-seq and RNA-seq signal around Fib-H, Sericin 1, Fib-L and Bmdimm gene.

Supplementary Figure S5. FAIRE DNA sonication agarose gel electrophoresis

Supplementary Figure S6. Person correlation (PCC) of samples

Supplementary Figure S7. Differentially expressed genes (DEGs)

Supplementary Figure S8. Silk gland GO annotation Directed Acyclic Graphs

Supplementary Figure S9. BmE GO annotation Directed Acyclic Graphs

Supplementary Figure S10. GO annotation of silk gland and BmE Up-regulated DEGs

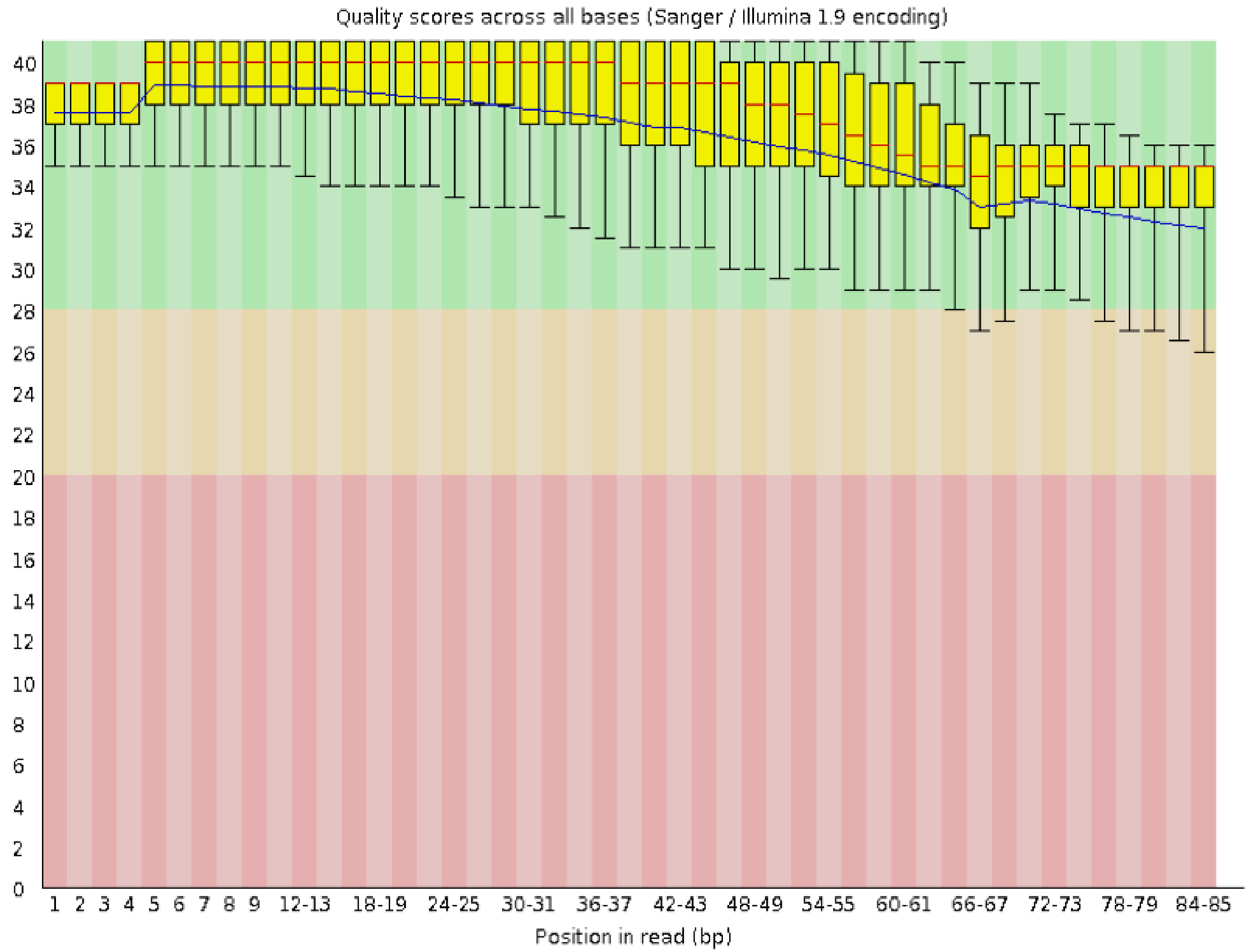

Supplementary Figure S1 Quality scores of all base. Almost all of the bases were high quality.

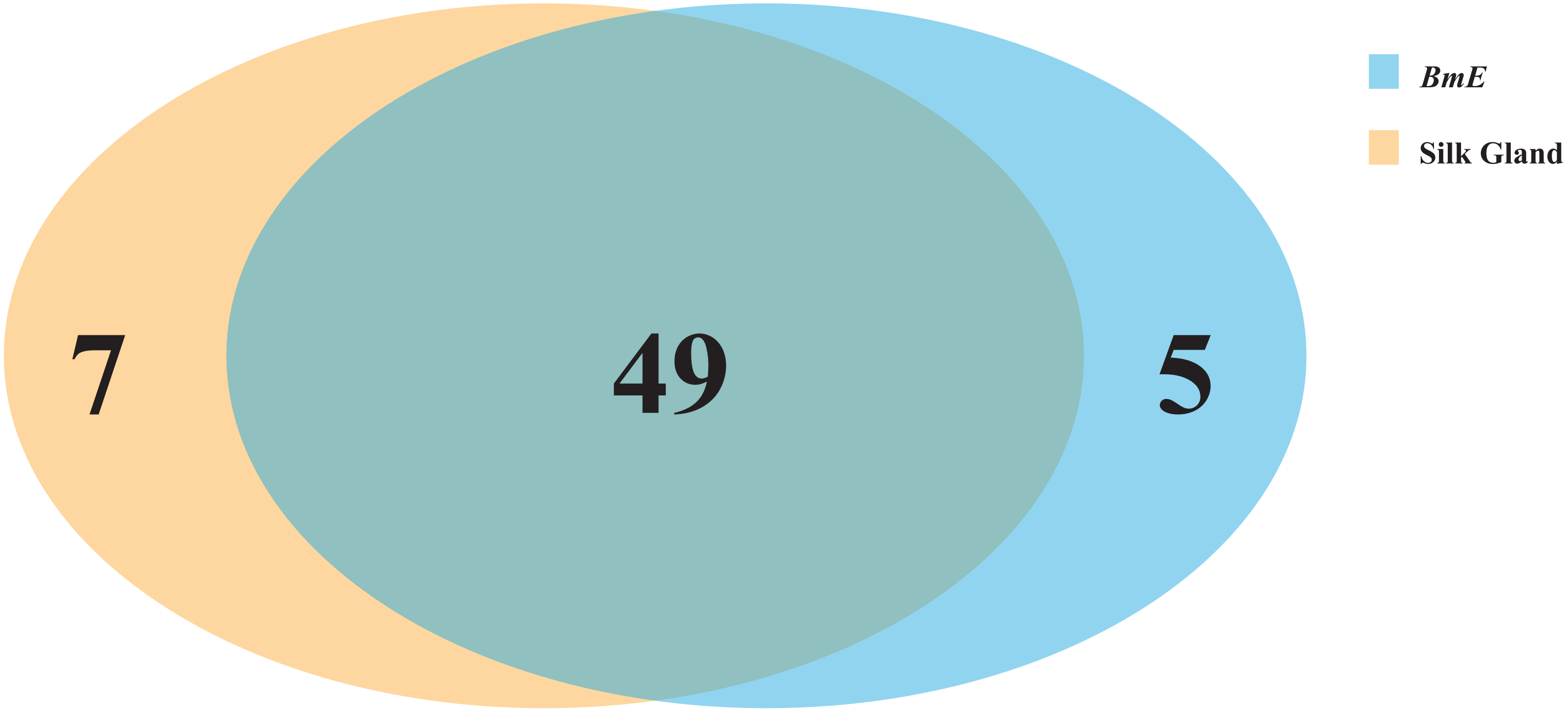

Supplementary Figure S2 venn graph of silk gland and BmE specific motifs. Blue was BmE and orange was silk gland.

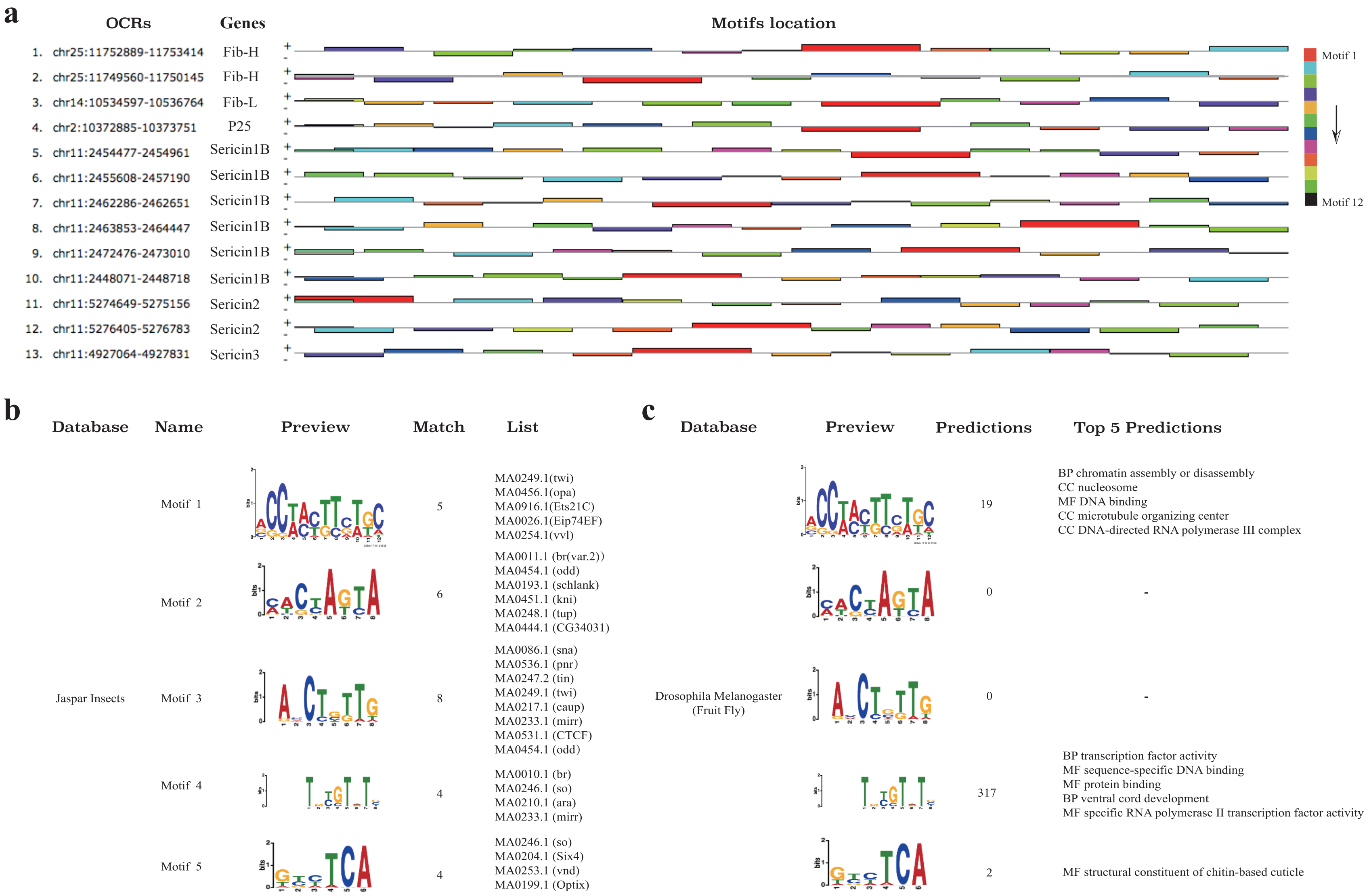

Supplementary Figure S3 A. Location of the new motifs in the open chromatin regions (OCRs). Different colors show different motifs. B. Known motifs similar to the newly discovered motifs. Here, we chose the E-value of the top 5 motifs. C. Gene ontolog annotation of the E-value of the top 5 newly discovered motifs.

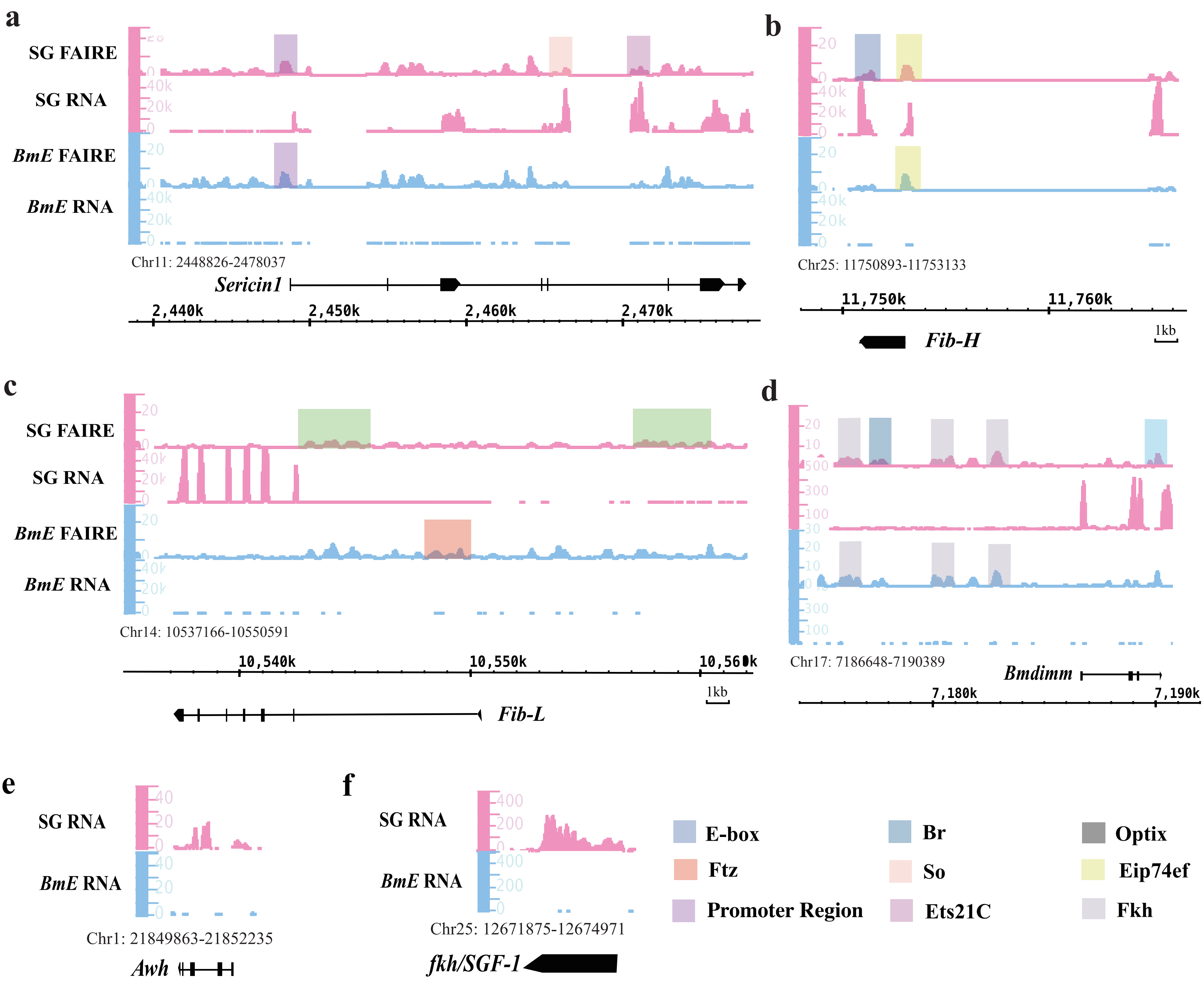

Supplementary Figure S4 FAIRE-seq and RNA-seq signal around Fib-H, Sericin 1, Fib-L and Bmdimm gene. Pink peaks are silk gland FAIRE-seq and RNA-seq peaks. Blue peaks are BmE FAIRE-seq and RNA-seq peaks. Other colored shadows show locations of known transcription factor binding sites, and one color corresponds to one transcription factor. A. Sericin 1. B. Fib-H. C. Fib-L. D. Bmdimm. E. RNA-seq signal peaks of Awh. F. RNA-seq signal peaks of SGF-1.

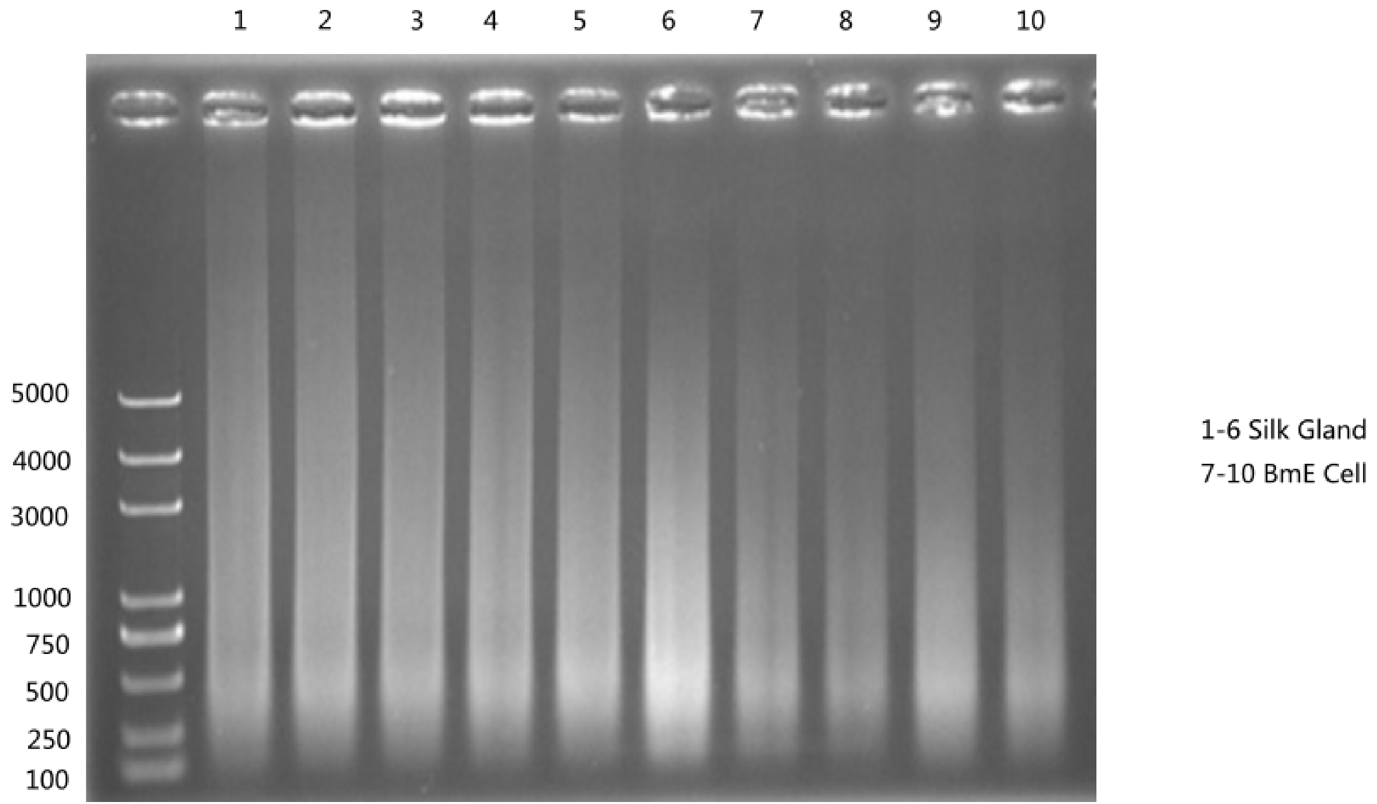

Supplementary Figure S5 DNA sonication agarose gel electrophoresis. The quantity contronl of FAIRE samples, 1,2,3,4,5,6 were the sonic crushing report of silk gland. 7,8,9,10 were BmE cell's. It indicated that the quantity of sonic crushing was very good, the main regiaon of DNA was enriched in 250-500bp.

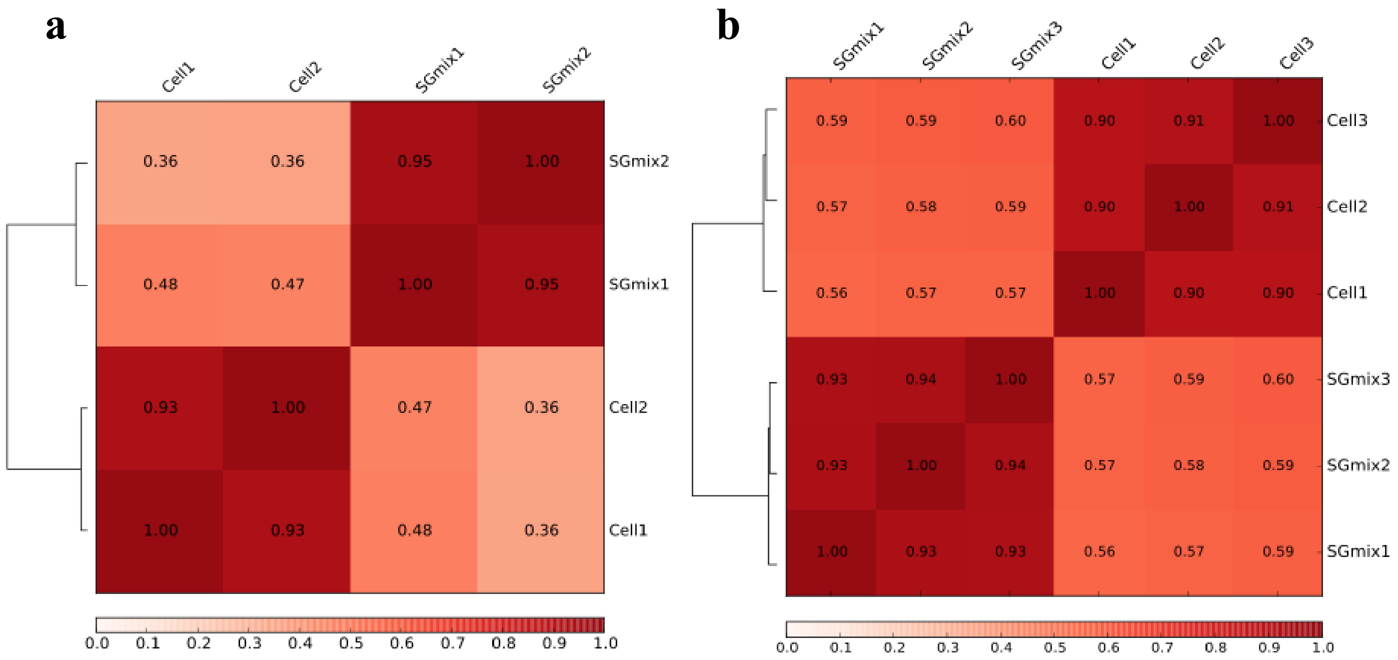

Supplementary Figure S6 Person correlation (PCC) of FAIRE-seq and RNA-seq samples. The color from light to dark indicted that the PCC was from low to high. Sample Cell was BmE cell, sample SG was silk gland. A. RNA-seq. B. FAIRE-seq.

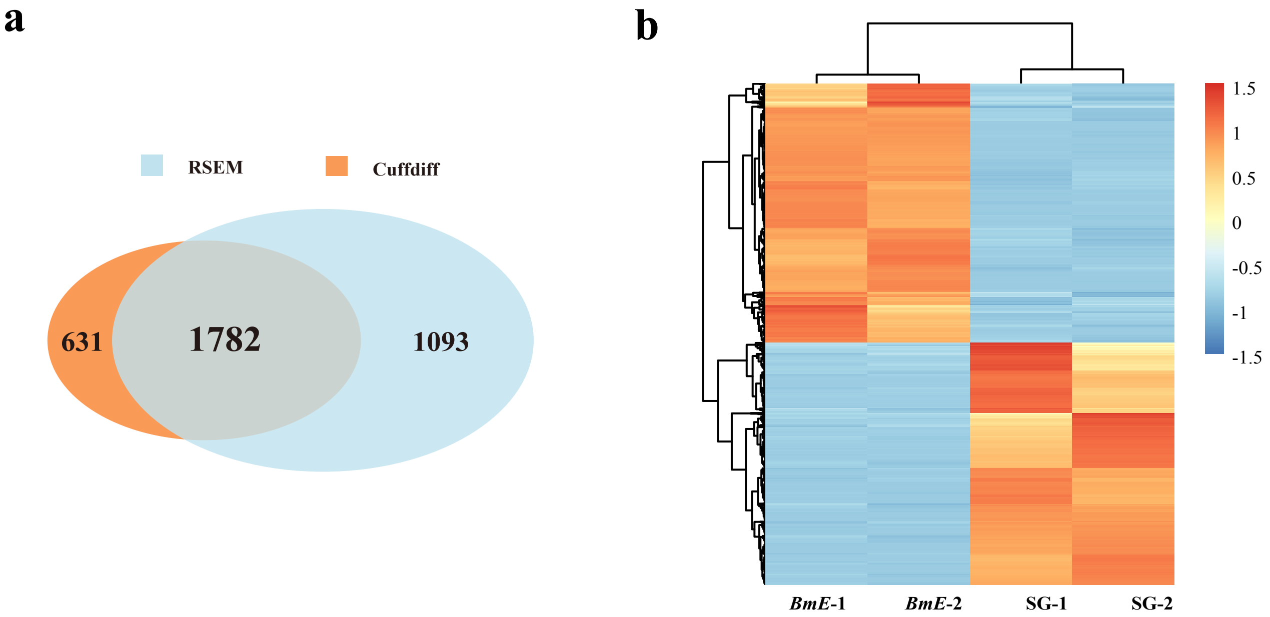

Supplementary Figure S7 Differentially expressed genes (DEGs) between silk gland and BmE. A. DEGs was generated by intersecting DEGs calculated by RSEM and Cuffdiff. The intersection was 1782. B. Heatmap of DEGs distribution in BmE and silk gland.

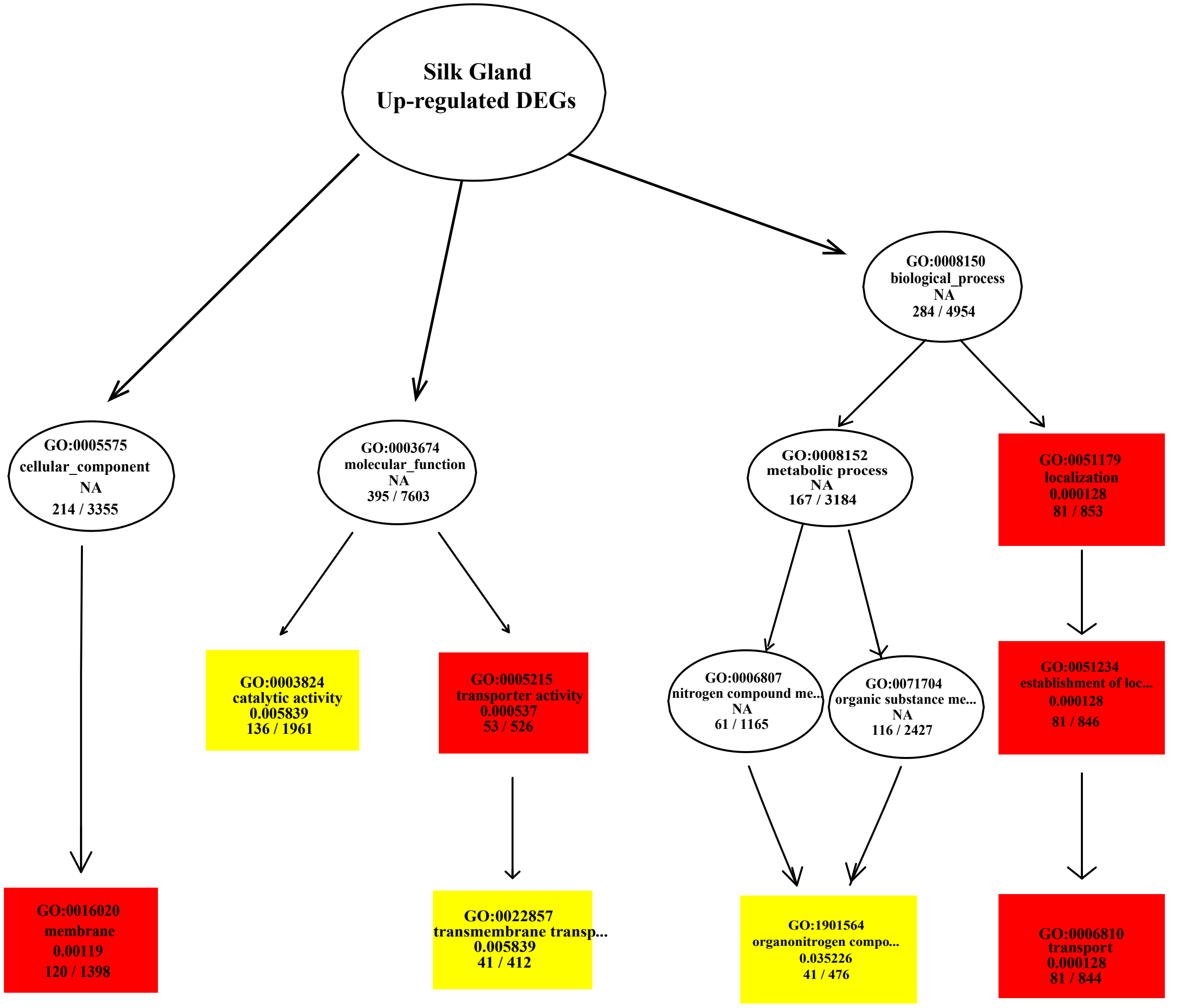

Supplementary Figure S8 Silk gland GO annotation Directed Acyclic Graphs. GO term in square was more remarkable than in ellipse. The red term was more remarkable than yellow. Each term was marked GO number, P-value, name and gene (DEG and background gene) number of enriched in this term. P-value <0.05 was remarkable.

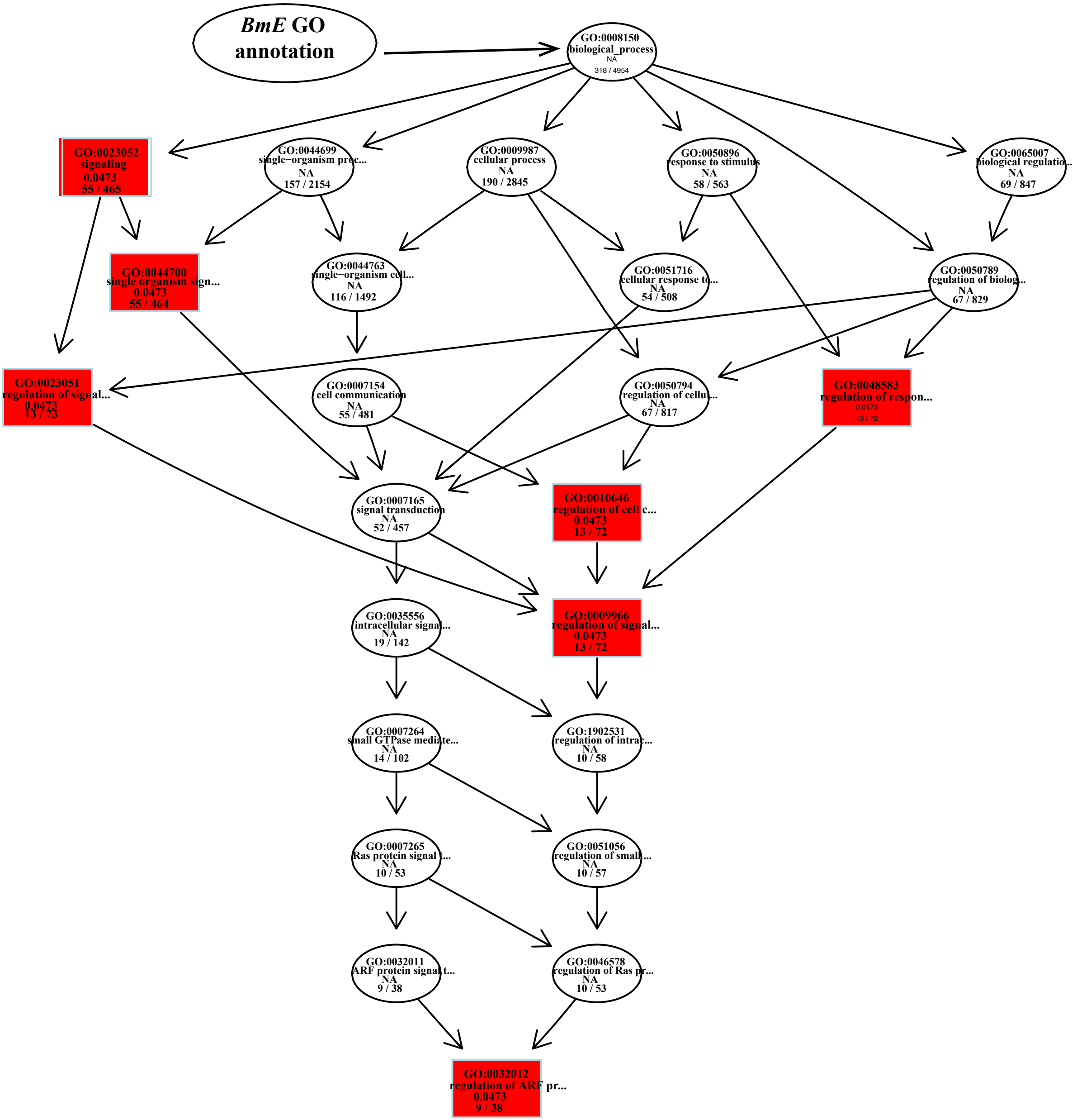

Supplementary Figure S9 BmE GO annotation Directed Acyclic Graphs. GO term in square was more remarkable than in ellipse. The red term was more remarkable than yellow. Each term was marked GO number, P-value, name and gene (DEG and background gene) number of enriched in this term. P-value <0.05 was remarkable.

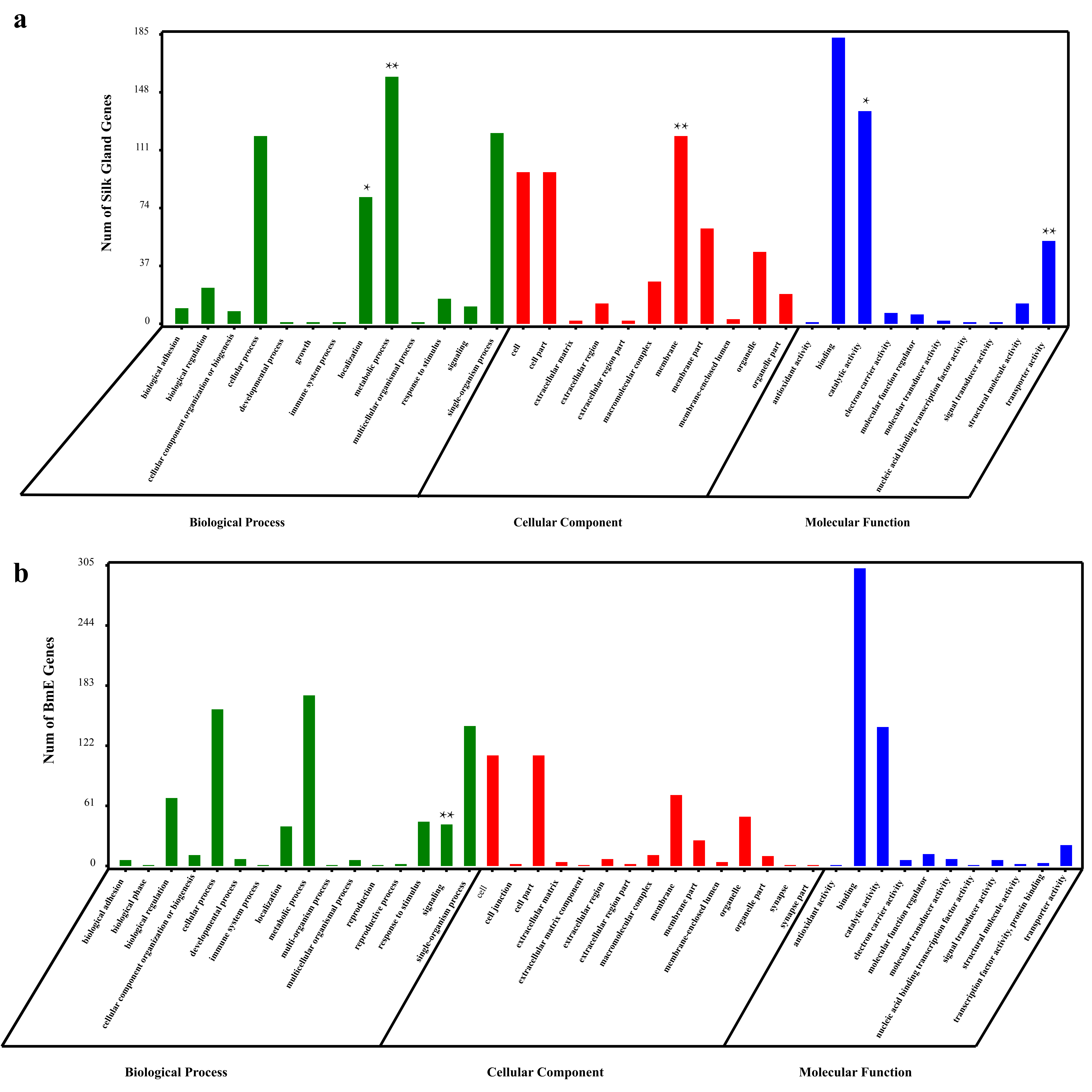

Supplementary Figure S10 GO annotation of silk gland and BmE Up-regulated DEGs. Asterisk mean that term was P-value<0.05 and it was the remarkable term. Two asterisk was the most remarkable term. A. Silk gland DEGs GO annotation. B. BmE DEGs GO annotation.
